# Adjustments of the Pesticide Risk Index Used in Environmental Policy in Flanders

**DOI:** 10.1371/journal.pone.0129669

**Published:** 2015-06-05

**Authors:** Davina Fevery, Bob Peeters, Sonia Lenders, Pieter Spanoghe

**Affiliations:** 1 Laboratory of Crop Protection Chemistry, Ghent University, Ghent, Belgium; 2 Flanders Environment Report, department Air, Environment and Communication, Flemish Environment Agency, Mechelen, Belgium; 3 Division for Policy Analysis of the department of Agriculture and Fisheries, Flemish government, Brussels, Belgium; University of Siena, ITALY

## Abstract

Indicators are used to quantify the pressure of pesticides on the environment. Pesticide risk indicators typically require weighting environmental exposure by a no effect concentration. An indicator based on spread equivalents (ΣSeq) is used in environmental policy in Flanders (Belgium). The pesticide risk for aquatic life is estimated by weighting active ingredient usage by the ratio of their maximum allowable concentration and their soil halflife. Accurate estimates of total pesticide usage in the region are essential in such calculations. Up to 2012, the environmental impact of pesticides was estimated on sales figures provided by the Federal Government. Since 2013, pesticide use is calculated based on results from the Farm Accountancy Data Network (FADN). The estimation of pesticide use was supplemented with data for non-agricultural use based on sales figures of amateur use provided by industry and data obtained from public services. The Seq-indicator was modified to better reflect reality. This method was applied for the period 2009-2012 and showed differences between estimated use and sales figures of pesticides. The estimated use of pesticides based on accountancy data is more accurate compared to sales figures. This approach resulted in a better view on pesticide use and its respective environmental impact in Flanders.

## Introduction

Pesticide use changes over time and its impact on human health and the environment is dependent on newly introduced pesticide products, climatic conditions, new resistant crop varieties and new scientific developments, such as formulation and spraying techniques. Pesticides are considered valuable and necessary to provide sufficient quantity of quality foods and to offer humans protection from vector-borne diseases. Pesticides can however give rise to a range of side effects such as health effects of the applicator, contamination of the water cycle, residues on agricultural products, toxicity for honey bees, birds, beneficial arthropods, etc. [1–2]. Due to the non-specificity of pesticides and losses during application, a portion of the applied pesticide ends up in non-target areas and organisms [2–5]. In Europe, highly polluting pesticides are prohibited since the 1970s (e.g. DDT). Other pesticides (e.g. lindane and parathion) are recently taken off the market under the review program of the European Union due to their eco-toxicity, endocrine disrupting effects or possible bio-accumulating properties [6–7]. The Environmental Policy and Nature Development Plan (MINA-plan) of the Flemish Government stipulates the objectives and principles of the Flemish environmental policy and provides the legal basis for a long-term policy on how to deal with the environment in a sustainable way [5].

An indicator based on spread equivalents (ΣSeq) is used in environmental policy in Flanders to quantify the pressure of pesticides on the environment. The sum of the annual spread equivalents per pesticide (ΣSeq) expresses the pressure that is caused by the use of pesticides on aquatic life. The use of each pesticide is weighted by differences in toxicity to aquatic organisms and residence time in the environment [5]. The ΣSeq, used since 1996, is included in the environmental policy of the Flemish Government for a regional evaluation of pesticide use [8]. In its 2003–2007 Environmental Policy Plan, the Flemish Government planned to reduce the pressure exerted by pesticides on aquatic organisms (expressed as ΣSeq) by 50% compared to the reference year 1990 [9]. That goal was shifted to 2010 in MINA-plan 3+. The MINA-plan 4 (2010–2015) stipulates a further decline in the coming period [7].

### 1.1 Sales and use of plant protection products

According to the European commission, pesticides include plant protection products and biocides [10]. Pesticides evaluated in this paper however only include all substances described as plant protection products (PPPs) in Belgian legislation [11]. These products are applied on plants as crop protection products. The term plant protection products (PPPs) will be used throughout this paper.

The determination of the exact amount of PPP usage in Flanders is difficult because sales figures are registered at a federal level (Belgium). Hence, the use of each product in the various crops and/or application areas is not sufficiently known. Depending on local growing conditions, cropping systems (indoor and outdoor) and recommendations of agronomists, the use of PPPs may vary significantly from area to area. Furthermore, the distribution of crops across land areas varies, even in time [7]. Based on Belgian PPP sales figures provided by the Federal government, the use of PPPs in Flanders was calculated, taking into account the ratio of crop areas for agriculture and the population number for non-agricultural use. This method is described in De Smet & Steurbaut [8]. For all products that were made available on the market after 2002, a method was developed to divide the quantities sold across all crops based on data from the Farm Accountancy Data Network (FADN), information provided by the official Belgian website displaying authorized plant protection products (Fytoweb), the percentage area of crops relative to the total crop area and per crop the ratio of the area in Flanders relative to Belgium [7].

Up to now, the use of PPPs (kg/year) in Flanders which is divided into groups (e.g. insecticides, fungicides and herbicides), target (agriculture, horticulture, non-agriculture) or crop group, is estimated based on sales figures from the Federal Public Service Health, Food Chain Safety and Environment (FPS) [8]. These data include the amount of active substances and not the commercial formulations, which contain all sorts of additives (including solvents, surfactants, and fillers). However, by stock processing, export and import, the actual use can be deviated from the sales figures [7]. This paper wants to address this difference in the framework of making policy decisions.

In 2010, sales figures that take into account the export of PPPs were provided for the first time by FPS. By using the corrected figures taking export exchange into account, a more accurate image of PPP usage in Belgium was obtained [7]. However, to better reflect reality, agricultural PPP use should be reconsidered. This PPP usage can be obtained by the Flemish FADN. The Farm Accountancy Data Network (FADN) exists for over fifty years now and is an EU-wide instrument for evaluating the income of agricultural holdings and the impact of the Common Agricultural Policy. Each year, accountancy data are collected from a representative sample of agricultural holdings in the European Union (EU) [12]. Until approximately 2005, only monetary values were registered in the Flemish FADN by the Centre for Agricultural Economics (CLE), e.g. the costs of PPP purchase. When more knowledge of the amount of PPP usage was required, additional studies had to be performed. As these studies implied a lot of paperwork, they were only performed for a few crops per year [13–15]. After computerization of the FADN in 2006 (this coincided with the split of the Centre for Agricultural Economics into the Social Sciences Unit, which was merged into Institute for Agricultural and Fisheries Research (ILVO), and the division for Policy Analysis, under the department of Agriculture and Fisheries of Flemish government), the monetary accounts were extended with an environmental module. That is how it became possible to register not only the costs, but also the quantities of PPPs purchased and stored on a farm. Farm PPP use per year is calculated as purchase plus stock on the first of January minus stock at the end of December. The annual use of PPPs of some 700 farmers is since then monitored yearly [7]. The area of non-agricultural use is not well researched. Since 2004, public services in the Flemish region have been reducing their PPP usage with the aim of obtaining a zero-use of PPPs by 2015. Each municipality and several public services (transport services, universities, etc.) annually report their PPP use to the Flemish government. Since 2012, information on non-agricultural use will become available due to the implementation of a separate registration of active substances for non-agricultural amateur use [16].

### 1.2 Objectives

The objective of this study was to modify the currently used Seq-indicator in order to better reflect reality. Total PPP use estimates, in this case estimates based on PPP sales, were compared to estimates based on usage registration like FADN. Accurate usage estimates are essential to all pesticide risk indicator calculations. In addition, this research refined and updated the Seq-indicator on three different aspects. First, the assessment of the distribution of the quantities sold in agriculture and non-agriculture was improved. Different crops in agriculture were reconsidered. This allows a better assessment of the different croppings regarding pressure on the water compartment. Second, the impact of the application method of the PPPs was included. Finally, the most recent toxicity data based on new European authorizations were processed in the calculation of the indicator.

## Materials and Methods

### 2.1 Description and application of the Seq-indicator

ΣSeq is an indicator that estimates the pressure of PPP use in both the different arable and horticultural crops as well as the pressure of the non-agricultural use on aquatic life. This indicator is calculated based on the following formula [5,7]:

$$\Sigma Seq\;=\;\frac{E\;\times {DT}_{50}}{MAC}$$

E = Annual sales of PPPs (kg active substance/year)

DT_50_ = Degradation time of 50% of the active substance in the soil (years)

MAC = Maximum Allowable Concentration (mg/l)

ΣSeq is a single-impact indicator that estimates the pressure exerted on one environmental compartment, i.e. the aquatic life (algae, daphnia, fish) [17–20]. However, the Seq-indicator only estimates the risk to aquatic organisms and does not take into account possible bio-accumulative capacity, potential endocrine disrupting characteristics nor synergistic effects. In addition, the indicator is only suitable for regional assessment and the handling of the Code of Good Agriculture Practice is not taken into account. The use of more appropiate formulation methods, certain cultivation techniques and strict compliance guidelines concerning the cleaning and rinsing of PPP tanks play an important role in reducing the burden of surface waters. Yet, these elements are not reflected in this indicator. Other indicators may display specific emission scenarios (SEPTWA, [21]) or estimate the risk for multiple components (POCER, [22]). Still, these indicators do not include the amount of annual used active substances [5,8].

### 2.2 Estimation of total regional/countrywide PPP use

The Federal Government (FPS) provides the Belgian sales figures for agricultural use on farms, while Belgian non-agricultural sales figures are provided by the industry (Phytofar). Sales figures are not available on regional level; furthermore, use figures in Flanders have to be combined from FADN (agricultural use estimates) and the Flemish Environment Agency (VMM, non-agricultural use). Finally, agricultural use of PPPs also includes seed treatment. Since the available data provide no information on seed treatment, seed treatment is considered a separate group under agriculture.

Up to 2012, the total use of PPPs has been seen as the sum of active ingredients, adjuvants and biological products. Additives and wetting agents are not effective themselves, but contribute to the active substance with a better ‘targeting’ as result [8]. For this study, the University of Ghent decided to include only chemical products based on the European list out of Annex III from the regulation of the European Union [23]. Beside the chemical products, some biopesticides registered by FADN that were not found in the European list [23], were included in the calculation. It concerns biopesticides listed in Annex II of the EU-regulation [24] and authorized in Belgium [25], i.e. mint oil, piperonylbutoxide, granulose virus, 1-dodecanol, 1-tetradecanol, potassium salts of fatty acids and paraffin oil (high & low sulf. Index). Every year the PPP list had to be updated, as some active substances are banned and new ones are added.

### 2.3 Agricultural use and sales of PPPs in different crops

Reconsidering the distribution of the quantities of PPPs sold in Belgium was twofold. Firstly, PPP use in different crop groups was based on FADN. Secondly, although the agricultural and amateur use of products was split following the new legislation [16], the non-separated sales figures still had to be used in this paper, since the division was not realised for the years studied here yet.

De Smet & Steurbaut [8] classified agriculture in thirteen crop groups. A fourteenth group was added, i.e. the pulses. Dry harvested pulses used to taken up in a separate group under agriculture in the context of the indicators of soil balance [26]. A fifteenth group of green manure was also added. Horticulture represents fruits, vegetables and ornamental crops in field and in greenhouses. The cultivation of potatoes, beets, maize, cereals, industrial crops, fodder, meadows and pasture, pulses and green manure is referred to as agriculture.

#### 2.3.1 Sales figures of PPPs collected by FPS

In Belgium, the federal government (FPS) annually requests the sales figures of PPPs from companies [8]. Nowadays, the Belgian sales figures are available from 1979 to 2012. The distribution of the quantities sold in agriculture and non-agriculture were reconsidered according to the following method. This method based on sales figures multiplies the FPS sales figures with the fractions of agricultural use on farms, seed treatment and non-agriculture. These fractions were estimated based on available data from FADN, industry (Phytofar) and VMM. This is illustrated by an example for the active substance of sulfur.

- The quantities of sulfur used in an agricultural context on farms, seed treatment and non-agriculture were divided by the total estimated use. The sum of the fractions equates to 3.9% (2.7% on farms, 0% in seed treatment and 1.2% in non-agriculture).
- The fractions were rescaled to 100%: 69% of sulfur was used on farms and 31% in non-agriculture.
- These obtained fractions were multiplied by the sales figures (FPS) of sulfur. These figures were used to determine the ΣSeq that is further on in this paper referred to as ΣSeq-value characterized by ‘method according to sales’.

#### 2.3.2 Use figures of PPPs collected by FADN

The division for Policy Analysis of the department of Agriculture and Fisheries provided data on the use of each product per crop for the years 2007 to 2012. Data concerning active substances used in PPPs are registered by the Farm Accountancy Data Network. The following numerical data were calculated and delivered for this study: the number of observations, the applied amount of active substance, the area of cultivation group and a weighted average expressed in kg of active substance per hectare of a cultivation group.

The data obtained from the Department of Agriculture and Fisheries were calculated based on several assumptions. The list of active substances registered in the survey included 378 substances of which 109 substances were not listed in the European list [23]. These 109 substances were excluded from the European list but still registered in the farmers’ use and therefore included in this study. Not included in this evaluation are organic farms, biological PPPs with an amount of zero use, products applied on animals, active substance/crop combinations that are prohibited—however, as a total herbicide is often used for the destruction of the ground cover plants before sowing of the crop, the use is assigned to the following crop according to FADN guidelines—and outliers on the total amount of active ingredient per hectares of maize, potato, beet and cereals. If holdings per year were insufficient to estimate a representative figure of the agricultural use of a PPP in a crop, the weighted average over the entire period (2007–2012) was included where possible. Outliers were defined as average uses larger than four times the standard deviation [27]. Areas of the different crop groups required to express the obtained data of FADN in kg active substance were provided by Statistics Belgium (DGSEI). Data from FADN were delivered on both regional (Flanders) and national (Belgium) level. The conversion factors (ratio of the areas) for various crops were based on the relationship between the growing areas per crop type in Flanders and Belgium (DGSEI). These factors were used to transform the use estimates of PPPs from Belgian level to Flemish level.

#### 2.3.3 Seed treatment

The process of applying fungicidal and/or insecticidal seed treatment products onto various types of seed as a protective coating to create a ‘protective zone’ of active substance in the soil against soilborne pathogens and insects is called seed treatment. Depending on the market requirements, a combination of different seed treatment products (fungicides and insecticides) is normally applied at varying application rates [28–29]. Treated seed entails less emission to surface waters [30–31], but they also pose certain risks, such as contamination of the environment by the emission of abraded seed particles during sowing [29]. Birds may also eat treated seed, which poses a serious risk to their health [22].

The seed treatment data were calculated by using various assumptions. For various active substances, desk research was conducted to determine whether they could be applied as seed treatment formulation. The provision of data for seed treatment was based on the products authorized in 2013 in Belgium. Seed treatment is used in many crops. These crops can be divided into four major groups:

- Vegetables: vegetables, peas, onions, shallots, leek, lettuce, endive, carrots and cabbages
- Cereals: (winter) wheat, maize, triticale, (summer and winter) barley, rye, oat, spelt
- Industry: chicory, flax
- Beet

The crop areas of Belgium were taken into account to quantify the amount of PPPs used in seed treatment. The amount of seed used per crop and the amount of active ingredient (dose/kg seed) required of that specific product to treat this seed were determined by using the seed quantity sown per hectare per crop. The amount of seed per crop was taken from Lenders et al. [26]. The amount of seed (per hectare) for wheat, maize and barley was determined by taking the average of respectively winter and summer wheat, grain and fodder maize, and winter and summer barley. In order to determine for certain products the exact amount of active ingredient, the parameter ‘number of seeds per hectare’ was needed. This number was obtained from Pannecoucque et al. [32] or calculated from the seed quantity expressed in kilograms per hectare using the number present in one gram. Specific values used for onions, leek, lettuce, endive and cabbage were based on data which were found in various sources [33–37]. The seed quantities expressed in ‘kg seed per hectare' and 'number of seeds per hectare’ used for the year 2009 are shown in Table 1.

**Table 1:** Seed quantities (kg/ha, #seeds/ha) and crop areas (ha) of various seeds treated crops in Belgium for the year 2009.

**Crop**	**Seed quantity (kg/ha)** *	**Number of seeds (#seeds/ha)** **	**Area Belgium (ha)** ***
Vegetables	10		40 940
Peas	10		9 200
Carrots	10	1 800 000	3 761
Onions	10	1 625 000	1 658
Shallots	10	1 625 000	6
Lettuce	10	12 500 000	181
Endive	10	7 000 000	84
Leek	10	3 700 000	3 383
Wheat	200		209 331
Winter wheat	175	3 500 000	206 281
Barley	160		44 810
Winter barley	135	2 500 000	40 512
Summer barley	185		4 298
Spelt	178		9 562
Rye	180		459
Oats	150		4 876
Maize	32.5	115 000	238 844
Triticale	165		6 666
Chicory	10	160 000	8 126
Flax	60	2 000 000	11 048
Beet	5	90 000	63 206
Cabbage	10	4 500 000	6 061

* [26]

** [32–37]

***[39]

The obtained quantities of active substance for seed treatment were multiplied by a fraction representing the average number of seed treatments across the total crop. These confidential data were obtained via experts of industry (Phytofar), phytofar group manufacturers and formulators of PPPs (phytosanitary or phytopharmaceutical products). The way in which data were provided by Phytofar is illustrated by an example based on data from Dutry [38]. According to this article, 50% of all pea seeds are treated with neonicotinoids (seed treatment factor of 0.50). As concerns winter barley, even two-thirds of all seed is treated. The calculation of the amount of PPP on treated seed is illustrated here with peas. According to Fytoweb, peas are treated with cymoxanil, fludioxinil, metalaxyl-m and thiamethoxam. Only thiamethoxam is a neonicotinoid and got the seed treatment factor of 0.50 [38]. This factor was multiplied by the seed quantity of 10 kg/ha, the Belgian peas area of 9 200 ha, the product dose of 0.15 l/100 kg seeds and active substance concentration of 350 g/l. Therefore, the amount of thiamethoxam in seed treatment of peas in Belgium was 24 kg. Following this method, the amount of seed treatment was calculated for each active substance and for all the crop groups.

The conversion of the national seed treatment data to the Flemish level was done by using the ratio of the cultivation areas of each crop group in Flanders and Belgium (DGSEI). Subsequently, the conversion factors for the various crops for the period 2009–2012 were calculated under the assumption that the part of treated seeds used in Wallonia equals the part of treated seeds used in Flanders.

### 2.4 Non-agricultural use of PPPs

Non-agricultural use of PPPs in Flanders so far was calculated on the basis of population ratio Flanders/Belgium. The population database could be retrieved from the DGSEI website [8]. In this study however, data for non-agricultural use were estimated by another way and based on two data sources. First, confidential national sales figures of amateur use were provided by industry (Phytofar). These sales figures apply to Belgium. Second, several data were recorded by the Flanders Environment agency (VMM). In the context of the project ‘Zonder is gezonder’ (2004), an online inventory was put in place: each municipality has to register its PPP use since then [40]. These data only apply to Flanders and are available for the period 2010–2012.

A conversion (CF) was performed by the amount used on the level of non-agricultural use in Flanders to the level of non-agricultural use in Belgium. Both surface area and use were charged (

Table 2). Use by municipality in Wallonia and Brussels, the other two regions in Belgium, was determined using the following formula:

$$Use\;in\;Flanders\;+Use\;in\;Wallonia\;+Use\;in\;Brussels\;=\;Total\;use\;in\;Belgium\;Use\;in\;Flanders\;+\;\left(Use\;in\;Flanders\;\times CF\right)\;+\;\left(Use\;in\;Flanders\;\times CF\right)\;=\;Total\;use\;in\;Belgium$$

**Table 2:** Conversion of data by public services of regional level (Flanders) to Wallonia and Brussels based on surface area (km^2^) and use (kg/year).

	**Surface area (km** ^**2**^ )*	# Municipalities*	**Surface area (km** ^**2**^ **/ municipality)**	**Conversion factor (CF)**	**Use**_**region**_ **(kg/year)**	**Use**_**municipality**_ **(kg/year)**
Flanders	13,521	308	44	1.00	**15,143** ***	49
Wallonia	16,844	262	64	1.46	22,108**	84
Brussels	161.38	19	8	1/5.17	2,929**	154

* [39,41]

** Use in Wallonia and Brussels was extrapolated from use in Flanders based on surface area per municipality

*** Known data from VMM

The overall use of PPPs in non-agriculture was determined by taking the sum of data obtained through Phytofar (Belgian level) and data obtained through VMM (Flemish level), and then converted to Belgian use. Since the ΣSeq was supplied in the first place for Belgium, data of VMM were—in a first step—rescaled to Belgian use based on the number of municipalities and use per municipality. In order to estimate the share of non-agriculture in Flanders, Belgian usage figures (Phytofar sales figures + VMM use figures) were multiplied by the conversion factor of 0.38. This factor was estimated by taking the ratio of non-agricultural use in Flanders (VMM) to non-agricultural use in Belgium (data obtained by conversion to Belgium based on the number of municipalities and use by each municipality). This conversion factor has the same value for the period 2010–2012.

### 2.5 Introduction of a weighting factor

#### 2.5.1 Emission pathways to surface water

PPPs can migrate to the surface through different pathways. The total amount of PPPs emitted to surface water depends on the properties of the active substance, the formulation, the local topography and the climatic conditions. An important distinction can be made between direct losses and diffuse losses [7,42–43].

The term direct losses includes leaking storage tanks, spills when filling spray tanks, tank washings or pour out of surpluses. Pollution carried out locally or on a limited scale in the vicinity of the source. Diffuse losses occur after applying PPPs on a field and are spread over a large area. The identification of a diffuse source is more difficult than of a direct source. Diffuse losses include mainly runoff, volatilization, drift and drainage [7,21,44–45].

Only direct losses and drift were taken into account in this study. Data were insufficiently available to take the remaining three pathways runoff, drainage and volatilization into consideration since these pathways especially depend on the properties of the chemicals.

#### 2.5.2 Applications and emission factors

PPPs can be sprayed or applied via seed treatment or a number of other application techniques. The application method of a specific PPP can be consulted via the official Belgian website displaying the authorized PPPs (Fytoweb). The website was consulted and every active substance was studied for the formulation it belongs to and whether it was possible to express the formulation (spray or seed treatment) in terms of percentage of each active substance’s use.

The result of the above exercise led to a specific weighting factor related to the use of the active substances with a particular method of application. A link with the crop type seemed the most reliable method to attribute the percentage distribution of the application method between the active substances. A literature review was done to address the question how to include the application methods in the impact calculation. Different emisision factors related to various application methods were described [21,46–49]. Regarding direct losses, sources Claeys et al. [48], Beernaerts et al. [47] and Pussemier & Beernaerts [21] indicate that the parameters are within the same order of magnitude. As regards drift, the parameters found in Claeys et al. [48] and Adriaanse et al. [46] are higher than the parameters found in Beernaerts et al. [47] and Pussemier & Beernaerts [21]. The model used in Beernaerts et al. [47] and Pussemier & Beernaerts [21] is based on expert judgment and relies on the state of studies conducted for each particular transport pathway applied to the specific situation of Belgium, which was not the case in the other studies mentioned above. This explains the difference in parameters for drift. Pussemier & Beernaerts [21] describe in detail the most different emission factors for drift and direct losses based on the SEPTWA-model. SEPTWA is empirically built based on emission factors, which assess and determine the emission of PPPs in specific circumstances. This model allowed us to assign an emission factor to various application methods. Table 3 illustrates the fractions of applied dose (f_der_, f_dir_, f_condm_) of various application methods responsible for drift and direct losses according to the SEPTWA-model. This table shows also the required various surface fractions (f_sup_, f_dirsup_, f_m_) under specific circumstances to determine the emission factors. For direct losses, the value was 0.05 throughout the study. The default value for drift was 0.01, except for orchards where more drift was expected, the fraction was increased to 0.03. The 'worst-case' scenario was assumed for greenhouse (Low Volume Application).

**Table 3:** Parameters to determine the emission factors (%) of various application methods for drift and direct losses according to the SEPTWA model [21].

Application	Emissions	Fractions*		Emission factors (%)
**Spraying orchards**	Drift	f_der_ = 0.03	f_sup_ = 0.03	0.09
	Direct losses	f_dir_ = 0.05	f_dirsup_ = 0.05	0.25
**Spraying fields and meadows**	Drift	f_der_ = 0.01	f_sup_ = 0.01	0.01
	Direct losses	f_dir_ = 0.05	f_dirsup_ = 0.05	0.25
**Backpack spraying**	Drift	f_der_ = 0.01	f_sup_ = 0.01	0.01
	Direct losses	f_dir_ = 0.05	f_dirsup_ = 0.05	0.25
**Seed treatment, pheromones and granules**	Drift	f_der_ = 0.00	f_sup_ = 0.01	0.00
	Direct losses	f_dir_ = 0.01	f_dirsup_ = 0.05	0.05
**Non-agriculture**	Drift	f_der_ = 0.00	f_sup_ = 0.01	0.00
	Direct losses	f_dir_ = 0.00	f_dirsup_ = 0.05	0.00
**Greenhouses**		f_condm_ = 0.001	f_m_ = 0.16	0.008

*f_dir_ = fraction of applied dose that is responsible for direct losses;

f_der_ = fraction of applied dose that is responsible for drift; f_dirsup_ = fraction of the water that can reach the surface water; f_sup_ = fraction of land area covered by surface water; f_condm_ = fraction of applied dose carried with condensation in method m; f_m_ = frequency of use in method m

#### 2.5.3 Determination of weighting factors

The choice of the weighting factors for various application methods was based on the emission factors determined in Table 3. In the last step, the spraying of orchards—a particular application that includes fruit (outdoor)—was assigned a weighting factor of 1. The spraying of fields and pastures comprises the crops of potato, beet, cereals, vegetables (outdoor), maize, industrial crops, ornamentals (outdoor), fodder, meadows and pasture, pulses and green manures. The weighting factors for these application methods and backpack spraying got a value of 0.76. Applications in greenhouse cultivation, non-agriculture and seed treatment received the weighting factors 0.02, 0.15 and 0.76 respectively. According to the SEPTWA model, the emission factor for use in non-agriculture is zero. However, drift and direct losses play a role in the application of PPPs in non-agriculture. Therefore, the application in non-agriculture was assigned the same weighting as backpack spraying. The final weighting factors were used to calculate the impact of the use of PPPs in the respective crops and for non-agricultural purposes.

### 2.6 Overview of the Seq-indicator according to new methods

Until now, the Seq-indicator was determined based on the sold quantities of PPPs (kg active substance per year) which were obtained by FPS. The Seq-indicator has in the present study been calculated in two different ways by using the above described methods. A specific weighting factor (wf) was introduced in both methods to link the substances with a particular method of application. First, in order to determine the ΣSeq, the estimated quantities of used PPPs (kg active substance per year) obtained from FADN were handled. The term used in this paper for this ΣSeq-value is ‘method according to use’. Second, the sales figures of FPS (0) were used to determine the ΣSeq and supply the ΣSeq-value ‘method according to sales’.

a) Seq_use incl wf_: *Total use*_*incl wf*_ (*kg*) = *wf*_*ag*_ × *use*_*ag*_ (*kg*) + *wf*_*non-ag*_ × *use*_*non-ag*_ (*kg*) + *wf*_*seed*_ × *use*_*seed*_ (*kg*). $Seq_{use\;incl\;wf}\;=\;Total\;use_{incl\;wf}\left(kg\right)\times \frac{DT_{50}}{MAC}$. b) Seq_use:$Total\;use\;\left(kg\right)\;=\;use_{ag}\left(kg\right)+use_{non-ag}\left(kg\right)+use_{seed}\left(kg\right)\;Seq_{use}\;=\;Total\;use\;\left(kg\right)\times \frac{DT_{50}}{MAC}$_
a) Seq_sales incl wf_: *Total sales*_*incl wf*_ (*kg*) = *wf*_*ag*_ × *sales*_*FPS*_ (*kg*) × *use*_*ag*_ (%) + *wf*_*non-ag*_ × *sales*_*FPS*_ (*kg*) × *use*_*non-ag*_ (%) + *wf*_*seed*_ × *sales*_*FPS*_ (*kg*) × *use*_*seed*_ (%). $\;Seq_{sales\;incl\;wf}\;=\;Total\;sales_{incl\;wf}\left(kg\right)\times \frac{DT_{50}}{MAC}$ b) Seq_sales_:$Total\;sales\;\left(kg\right)=sales_{FPS}\left(kg\right)\times use_{ag}\left(\%\right)+sales_{FPS}\left(kg\right)\times use_{non-ag}\left(\%\right)+sales_{FPS}\left(kg\right)\times use_{seed}\left(\%\right)\;Seq_{sales}=Total\;sales\;\left(kg\right)\times \frac{DT_{50}}{MAC}$

### 2.7 Update of toxicity data

The residence time (persistence) of PPPs in the environment varies from several days to years. Soil half-life (DT_50_) is the time it takes for 50% of the compound to break down in the soil, by biological or physicochemical processes. The longer DT_50_, the more likely the PPP is to leach through the soil and contaminates water bodies [50]. The Maximum Allowable Concentration (MAC-value) is determined on the basis of six different toxicity values for the representative aquatic organisms, i.e. the acute or chronic toxicity to three trophic levels: algae, crustaceans and fish (EC_50algae_, NOEC_algae_, LC_50crustacea_, NOEC_crustacea_, LC_50fish_ and NOEC_fish_). The NOEC (No Observable Effect Concentration), concentration at which prolonged exposure has no observable effect on the test species, defines the chronic toxicity. The acute toxicity includes the concentration at which 50% of the test species cause mortality in a single dose (LC_50_, Lethal Concentration) or the concentration at which 50% of the test species cause a desired effect (not necessarily mortality) (EC_50_, Effect Concentration). Toxicity values for the same species are always chosen, as sensitivity within the same class may differ. If no data are available, the lowest available toxicity value is opted for. The available toxicity data are often incomplete and therefore a safety factor has to be taken into account. This stems from the precautionary principle—if toxicity values are missing—to absorb the differences in sensitivity to pollutants between different classes of indicator organisms. The less parameters for determining toxicity at different trophic levels are available, the higher the safety factor used [7,8]. These safety factors are derived from the recommendations for standards according to the European Water Framework Directive [51]. The MAC-values are calculated through dividing the lowest toxicity value by the safety factor [5,8]. Table 4 gives an overview of the safety factors needed to derive MAC-values of the toxic properties.

**Table 4:** Safety factors for derivation of Maximum Allowable Concentration (MAC) values of the toxic properties [52–53].

**Available data**	**Safety factor**	**MAC**
NOEC-values of at least 3 trophic levels (algae, crustacea and fish)	10	Lowest toxicity/10
NOEC-values of at least 2 trophic levels (algae, crustacea or fish)	50	Lowest toxicity/50
NOEC-values of 1 trophic level (crustacea or fish)	100	Lowest toxicity/100
Only NOEC-value of algae or just L(E)C50-values of aquatic species	1000	Lowest toxicity/1000

Previous work showed that especially the MAC-values in surface waters differ [5,8]. Since 2010, the University of Ghent disposes of a full revised database that includes the properties of all known (authorized and unauthorized) active substances. These data are in line with the new official data from the review program for PPPs in the EU. The following sources for parameter values, ranked as function of importance, were used to create the database (University of Ghent): authorization files of the EU, the Footprint database [54] and the database of UGent supplemented with new products until 2009 including the information of the Tomlin Pesticide Manual.

Concerning the priority substances 2,4-D, chloridazon, dimethoate, diuron, malathion, MCPA and mecoprop, parameter values were derived from background documents used for the deriving the Environmental Quality Standards for Priority Substances. The Fraunhofer Institute was appointed by the European Commission (EC) in order to set up these documents (Table 5). Please note that the minimum requirements were not in line with those of the Water Framework Directive [51].

**Table 5:** Maximum Allowable Concentration (MAC) values (mg/l) for the priority substances according to Fraunhofer institute different from EU authorization files and footprint database [54].

Active substance	Fraunhofer institute MAC (mg/l)
2,4-D	0.0185
chloridazon	0.0100
dimethoate	0.00002
diuron	0.0002
malathion	0.0000008
MCPA	0.0007
mecoprop	0.0130

## Results and Discussion

### 3.1 Use and sales figures of PPPs

**Fig 1:**
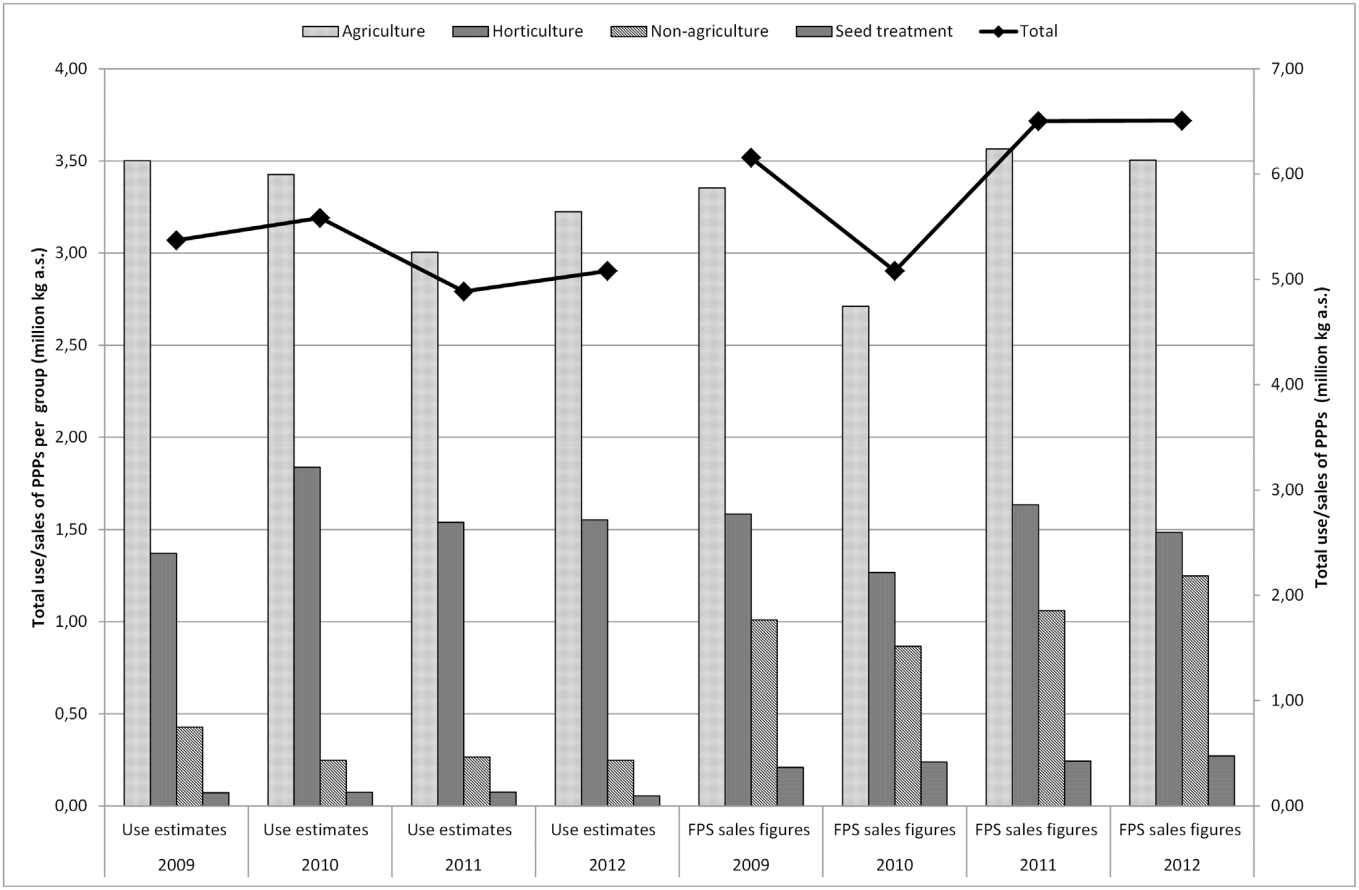
Total PPP usage estimates (million kg a.s.) obtained by Farm Accountancy Data Network (FADN), industry (Phytofar) and Flemish Environment Agency (VMM) and total PPP sales figures (million kg a.s.) obtained by Federal Public Service (FPS) per group (agriculture, horticulture, non-agriculture and seed treatment) in Belgium for the period 2009–2012.

**Fig 2:**
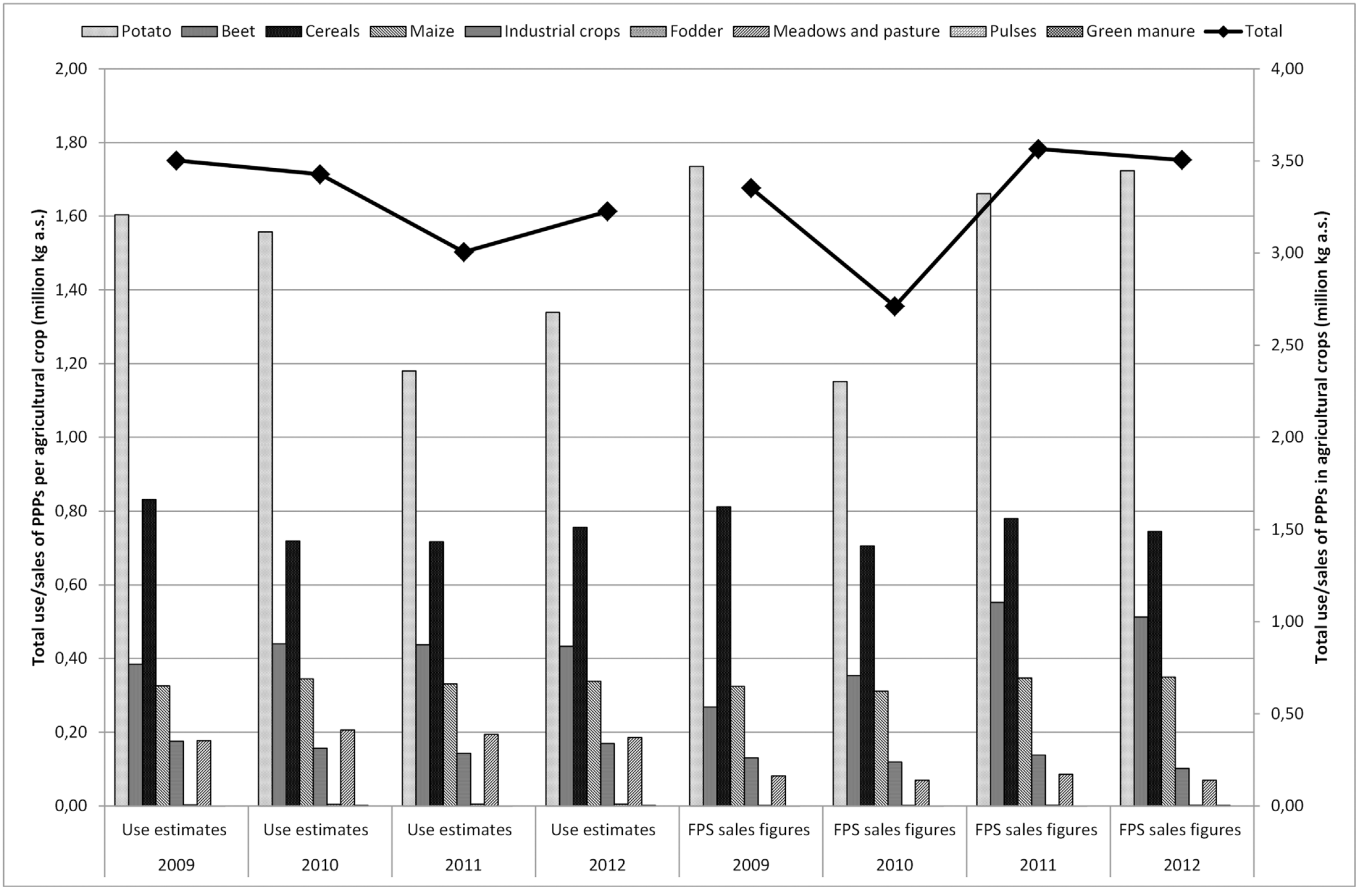
Total PPP usage estimates (million kg a.s.) obtained by Farm Accountancy Data Network (FADN), industry (Phytofar) and Flemish Environment Agency (VMM) and total PPP sales figures (million kg a.s.) obtained by Federal Public Service (FPS) per agricultural crop (potato, beet, cereals, maize, industrial crops, fodder, meadows and pasture, pulses and green manure) in Belgium for the period 2009–2012.

**Fig 3:**
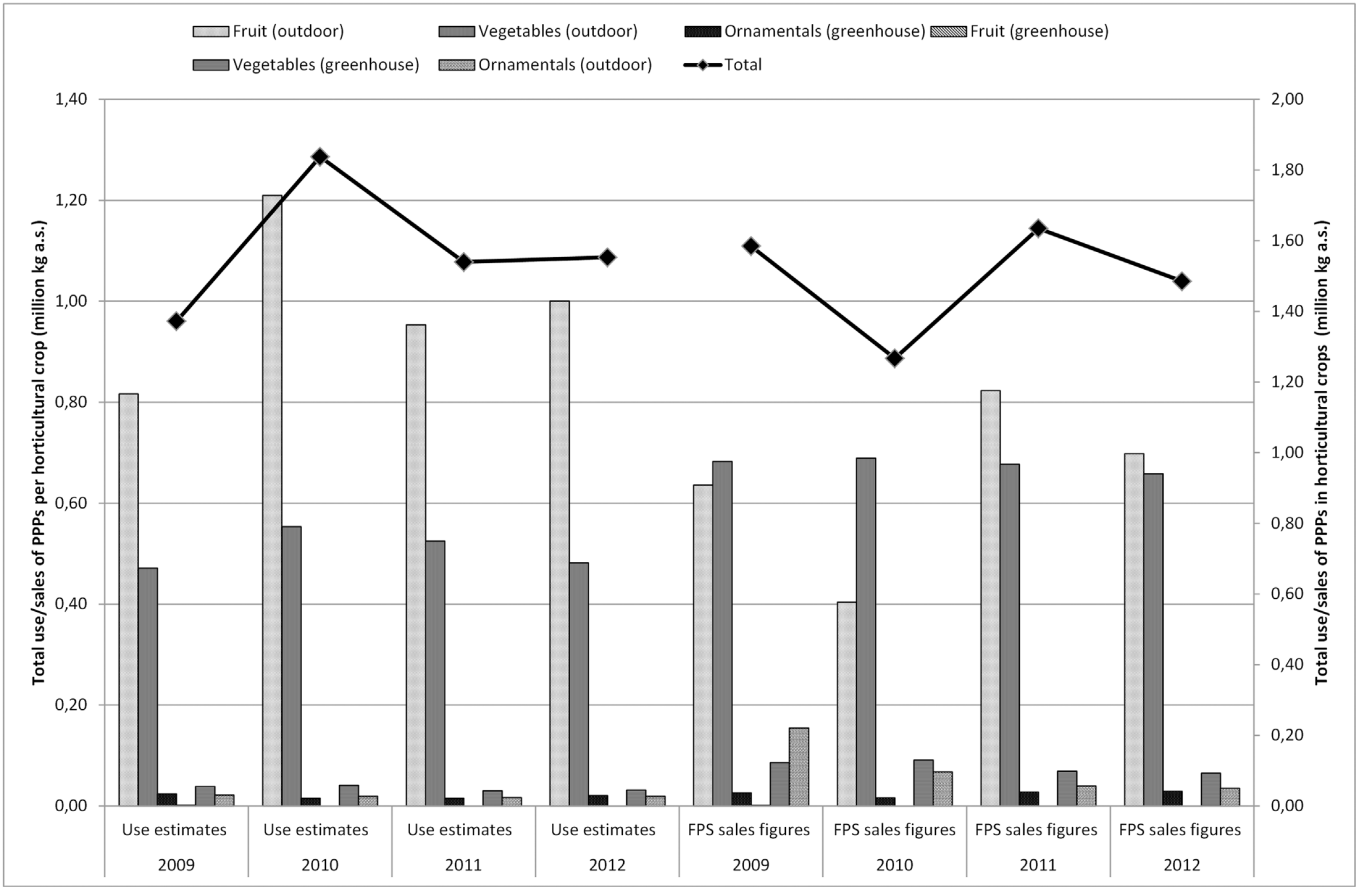
Total PPP usage estimates (million kg a.s.) obtained by Farm Accountancy Data Network (FADN), industry (Phytofar) and Flemish Environment Agency (VMM) and total PPP sales figures (million kg a.s.) obtained by Federal Public Service (FPS) per horticultural crop (fruits, vegetables and ornamental crops in field and in greenhouses) in Belgium for the period 2009–2012.

Figs 1 to 3 show a comparison of the estimated total Belgian PPP use based on purchases by farmers and amateurs or on total sales recorded by FPS. In order to modify the Seq-indicator, the objective of this research was to relate the national sales figures to a regional level (Flanders). The sum of the usage estimates of agricultural use on farms, seed treatment and non-agriculture should be comparable to the sales data of the national government (FPS). In general, Fig 1 shows a slight decrease in the use estimates of PPPs for the period 2009–2012 whereas the sales figures reflect a capricious pattern. For 2009, 2011 and 2012, use estimates of PPPs in all groups (except for agriculture in 2009 and horticulture in 2012) were lower than PPP sales. In 2010, the estimated use of PPPs for agriculture and horticulture was higher than sales figures whereas the estimates of non-agricultural use and seed treatment were again lower than sales figures. The trend between use and sales can be explained by Table 6, which summarizes the active substances with the largest influence on the total use estimates and sales of PPPs. For example, in 2009 the estimated use of mancozeb was 825,539 kg, while the FPS sales figures indicate that 1,189,363 kg mancozeb was sold in 2009. In 2010, the estimated use of mancozeb was higher than the sales figures while the use estimates in 2011 and 2012 were again lower than the sales. The use estimates of glyphosate were lower than the FPS sales figures throughout the period. In practice, PPPs purchased in a given year might not be used during that time of the year due to the non-presence of a particular disease or pest. Hence, it is logical that during a certain year the use is lower than sales would indicate. Then again, a proportion of the seed is also treated abroad and imported into Belgium. Please note that the import figures of PPPs on seeds are not taken into account in FPS’ sales figures. These seed treatments may have an impact on the environment in Belgium and Flanders. The difference between sales and use estimates can also be explained by economic reasons, such as budgets that need to be spent in one year or commercial actions recommending certain products which results in a stock of PPPs.

**Table 6:** PPP usage estimates (kg) obtained by Farm Accountancy Data Network (FADN), industry (Phytofar) and Flemish Environment Agency (VMM) and PPP sales figures (kg) obtained by Federal Public Service (FPS) of the active substances in Belgium with the largest impact on results for the period 2009–2012.

**Active substance**	**Use estimates (kg)**				**FPS sales figures (kg)**			
	2009	2010	2011	2012	2009	2010	2011	2012
Mancozeb	825,539	807,470	567,374	620,074	1,189,363	672,230	966,081	941,778
Glyphosate	340,584	334,168	360,808	384,538	434,797	371,465	552,861	699,387
****Total****	**5,372,250**	**5,585,301**	**4,885,710**	**5,080,962**	**6,156,252**	**5,080,852**	**6,568,943**	**6,510,267**

Fig 2 compares the estimated use of PPPs in Belgium in the major agricultural crops to the FPS sales figures. From this figure, it is clear that the total sales and use estimates largely follow the trend in the cultivation of potatoes. Besides the possible reasons mentioned in the previous section, sales and use of PPPs may also fluctuate annually depending on, for instance, the weather and the acreage [55]. Fig 3 shows the comparison of the use estimates of PPPs in Belgium in the main horticultural crops to the national sales figures. The graph especially follows the profile of the use in the fruit crop group. Striking here is that the use of PPPs for cultivation in greenhouses and ornamentals is hardly remarkable. The estimated usage of fruit (outdoor) is much higher than sales. The question here is whether the deleted statistical relevant crops with less than six response data by FADN caused any rupture of the trend. The total figures per year remain within the same order of magnitude. However, the division put more emphasis on the fruit whereas reality might be different. Special focus on collecting data on greenhouse cultivation and ornamentals in the future can improve representativeness.

### 3.2 ΣSeq-values

**Fig 4:**
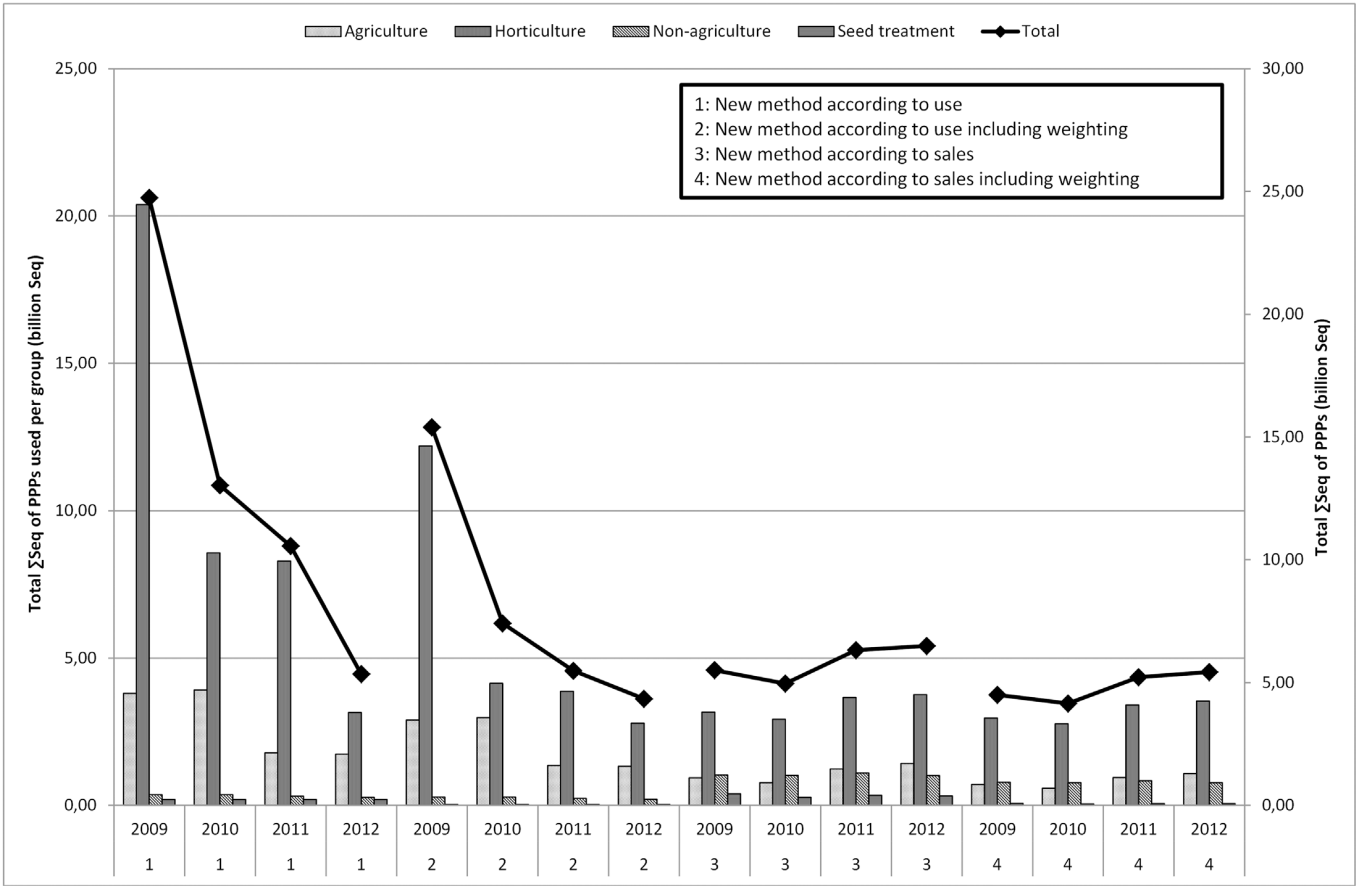
Total ΣSeq of PPPs (billion Seq) used in agriculture, horticulture, non-agriculture and seed treatment in Flanders for the period 2009–2012 by using the method based on usage estimates and based on sales figures (with or without weighting).

**Fig 5:**
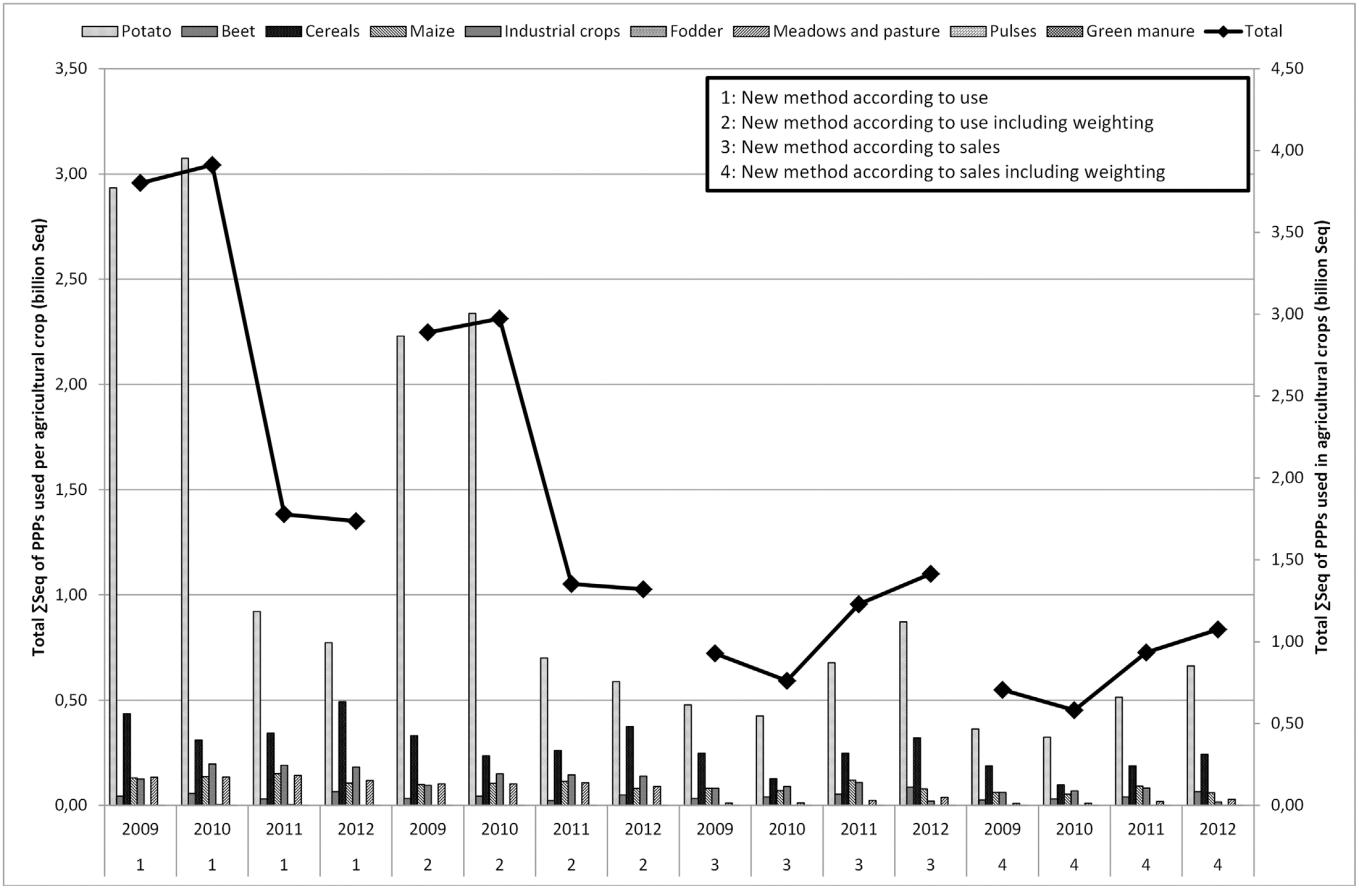
Total ΣSeq of PPPs (billion Seq) used in agricultural crops (potato, beet, cereals, maize, industrial crops, fodder, meadows and pasture, pulses and green manure) in Flanders for the period 2009–2012 by using the method based on usage estimates and based on sales figures (with or without weighting).

**Fig 6:**
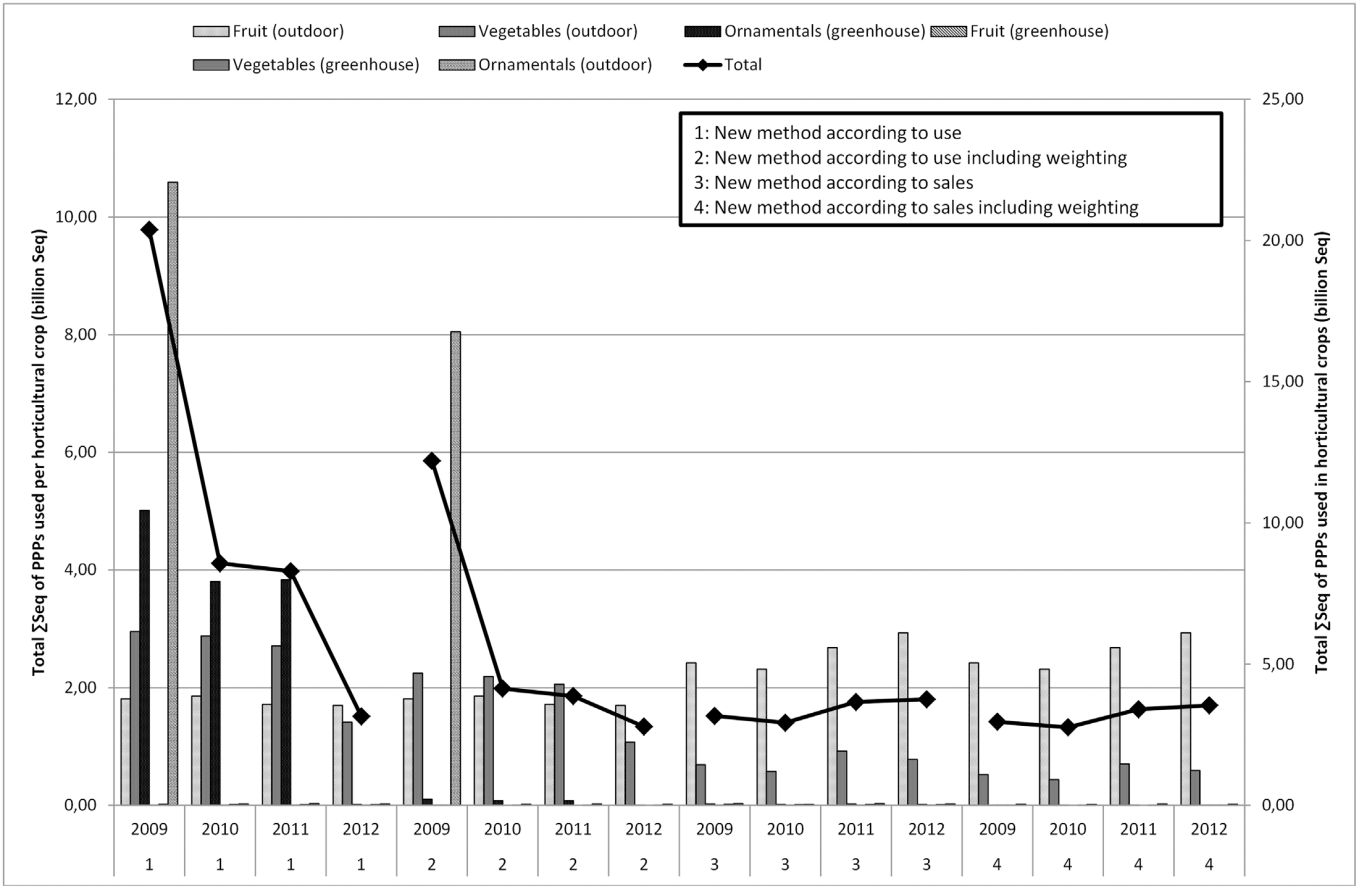
Total ΣSeq of PPPs (billion Seq) used in horticultural crops (fruits, vegetables and ornamental crops in field and in greenhouses) in Flanders for the period 2009–2012 by using the method based on usage estimates and based on sales figures.

Figs 4 to 6 present the ΣSeq-values for Flanders. The ΣSeq based on sales figures (determined by the percentage of use) is lower than the ΣSeq based on usage figures. Fig 4 describes the sum of ΣSeq for PPPs used in agriculture, non-agriculture and seed treatment by using the usage figures and the sales figures (with and without weighting). In Fig 4, a clear difference between method 1 & 2 (use and weighted use estimates) and method 3 & 4 (sales and weighted sales data) is noted. The lower ΣSeq-values obtained by using the method based on sales, can be explained by certain active substances still in use even if they were no longer authorized, sold or imported. Table 7 summarizes the active substances with the largest influence (sorted by year 2009) on the determination of the ΣSeq. For example in the period 2009–2011, endosulfan, fentin-hydroxide and paraquat were still used in Belgium whereas the national sales figures indicate that 0 kg of these active substances was sold. This leads to a ΣSeq-value (sales) of zero and explains why the ΣSeq-values based on sales show much lower values since not all active substances were taken into account. The results of the ΣSeq should be treated with caution. Although the sales of some active substances were no longer allowed in the period 2009–2011 (e.g. endosulfan), they were still used in small amounts. In 2012, endosulfan was no longer used in Belgium. Toxicity parameters also have a large impact on the ΣSeq-value, even if only a small amount of a certain active substance is used.

**Table 7:** National estimated use (kg) based on data from Farm Accountancy Data Network (FADN), industry (Phytofar) and Flemish Environment Agency (VMM) and sales figures (kg) obtained by Federal Public Service (FPS), ΣSeq based on use estimates and sales figures of the active substances in Flanders with the largest impact on results for the period 2009–2012.

**Active substance**	**Use estimates (kg)**				**FPS sales figures (kg)**				**ΣSeq**_**use**_ **(billion Seq)**				**ΣSeq**_**sales**_ **(billion Seq)**			
	2009	2010	2011	2012	2009	2010	2011	2012	2009	2010	2011	2012	2009	2010	2011	2012
Endosulfan	167	59	59	0	0	0	0	0	17.67	5.90	5.82	0.00	0.00	0.00	0.00	0.00
Fentin-Hydroxide	770	776	0	0	0	0	0	0	1.88	1.96	0.00	0.00	0.00	0.00	0.00	0.00
Paraquat	1,393	1,278	992	1,615	0	0	0	0	1.27	1.26	0.92	1.58	0.00	0.00	0.00	0.00
Koper-oxychloride	48,189	43,647	31,846	23,849	36,076	28,730	24,894	0	1.07	1.03	0.69	0.44	0.83	0.61	0.52	0.00
Koper-hydroxide	20,530	21,555	28,027	36,381	46,210	49,020	59,505	71,495	0.73	0.76	1.00	1.30	1.83	1.94	2.35	2.83
****Total****	**5.37E+06**	**5.59E+06**	**4.89E+06**	**5,08E+06**	**6.16E+06**	**5.08E+06**	**6.57E+06**	**6,51E+06**	**24.74**	**13.03**	**10.56**	**5.34**	**5.50**	**4.96**	**6.32**	**6.49**

### 3.3 Impact of weighting factors

The impact of the weighting factors in method 1 & 2 and method 3 & 4 is low. The effect of weighting factors according to the environmental impact of an application is more apparent when looking at different crops. The ΣSeq-value of the crop group agriculture, decreases between method 1 and 2 with approximately 25%. A decrease of approximately 25% is obvious, since only 76% of the estimated use is taken into account in ΣSeq calculations for agricultural crops when weighting is applied. Horticulture, including fruit, greenhouse cultivation and ornamentals, shows a decrease of about 40% due to incorporating weighting factors (the weighting factor is 0.02 for greenhouses, 0.76 for ornamentals and vegetables). Using different active substances in the ΣSeq every year can strongly influence the total ΣSeq per crop. Compared to 2009, the ΣSeq for ornamentals (outdoor), for example, decreases to almost zero in 2012 (Fig 6). As of 2010, endosulfan was no longer used in ornamentals (outdoor), which explains the decrease of the ΣSeq. The effect of the weighting factors between methods 1 & 2 or 3 & 4 in Fig 5 follows the same trend, as the same weighting factors are used for the different crops. The effect of the weighting factors is clearly visible in Fig 6. This figure shows ΣSeq-values of PPPs used in horticulture. The ΣSeq-value for ornamentals decreases tremendously due to incorporating a weighting factor of 0.02 assigned to the environmental pollution.

### 3.4 Discussion

Sales figures of PPPs recorded by FPS, are relatively simple to collect and fairly inexpensive. However, sales figures can give rise to confidentiality issues and restrictions on the release and use of data for commercial reasons. The FPS sales figures are not available on the active substance level and contain no information about the crop, timing, regional variation in use, dose applied, number of applications to the crop or percentage of crop treated (1.1). Usage estimates cover all kinds of data on the actual use of PPPs by farmers and growers, but are not always quick and easy to produce (2.3.2). On the other hand, reliable data on usage of PPPs are critical for the development of indicators of the effects of PPPs on the environment. As shown in Figs 1 to 3, the differences between sales figures and usage estimates of PPPs are not as impressive. However, the identified differences exert a large influence on the determination of the pressure on aquatic life (3.1, 3.2). It is acknowledged that use indicators (kg a.s.) are not adequate proxies for assessing pressure exerted by PPP use [5,8]. Furthermore, a more reliable distribution of PPP use among crops is established by using usage estimates instead of sales figures of PPPs. Figs 4 to 6 show significant differences between the ΣSeq-values based on sales and based on usage estimates. The ΣSeq-values based on sales show much lower values since not all active substances were taken into account. In addition, the toxicity parameters of the active substances also exert a significant influence on the results of the indicator. An active substance can be highly toxic to the environment even if only a small amount of the active substance is used (e.g. endosulfan). So accurate usage estimates and toxicity parameters of PPPs are essential to all pesticide risk indicator calculations to better reflect reality. In addition, sales figures may be used to adjust and improve surveys on use of PPPs. The application method was included into the risk indicator calculations based on weighting factors (3.3). Taking into account the weighting factors into the calculations of the ΣSeq, provides a strong reduction of the indicator.

The adjusted method applied to estimate the total use of PPPs was used for the period 2009–2012. Still, this period is too short to see a long-term evolution. In 2009–2012, agriculture and horticulture were responsible for approximately 95% of the total use estimates. The use of PPPs in arable farming was circa 50% larger than in horticulture. Throughout the time frame, the average for non-agriculture and seed treatment was respectively 3.5% and 2%. The most commonly used PPPs were fungicides and herbicides. In fact, a limited number of PPPs determine the total ΣSeq (Table 7). Horticulture had over the entire period the largest influence on the ΣSeq followed by agriculture, non-agricultural use and seed treatment. In this short period, the ΣSeq-value based on use estimates of PPPs declined between 2009 and 2012. This decline is particularly caused by a reduced use of endosulfan (insecticide, prohibited in 2007). The ΣSeq based on sales figures shows a pattern as capricious as sales figures (Fig 1). ΣSeq is a simple indicator that requires limited data input and provides an easy tool for environmental policy planning. However, it is necessary to complement the databases with new information and research results to ensure the transparency of the applied data and to avoid misinterpretations of the policy makers.

## Conclusion

The objective of this study was to modify the currently used Seq-indicator to better reflect reality. Total PPP use estimates, in this case estimates based on PPP sales, were compared to estimates based on usage registration. In general, this study showed the difference between use estimates and sales figures of PPPs. The estimated use of PPPs is more accurate compared to sales figures. Use estimates were lower than national sales figures and particularly followed the trend of cultivation of potatoes and fruit. The ΣSeq was calculated in two different ways: based on usage estimates and based on FPS sales figures. However, the ΣSeq-values determined by the method based on sales figures were much lower. A certain number of PPPs can be sold in a certain year, but are not necessarily applied in that year. This was clearly shown in this study. A PPP like endosulfan, fentin-hydroxide or paraquat, not registered in sales figures, were still in use and had a remarkable pressure on surface water. The ΣSeq-values based on sales show lower values, since not all active substances were taken into account. Another remark concerns toxicity parameters, which have a large impact on the ΣSeq-value, even if only a small amount of a certain active substance is used. Accurate usage estimates and toxicity parameters of PPPs are essential to the ΣSeq-indicator calculations to better reflect reality.

This research also refined and updated the Seq-indicator on at least three other aspects. First, the distribution of the quantities sold in agriculture and non-agriculture and the different crops in agriculture have been reviewed. The calculations were carried out based on usage figures of PPPs. Non-agricultural use and seed treatment data were also incorporated. Second, weighting factors were calculated to include the application method. Taking into account the weighting factors into the calculations of the ΣSeq, provides a strong reduction of the ΣSeq-value. Finally, the most recent toxicity data based on new European authorizations were processed in the calculation of the indicator.

Finally in the present study, non-agricultural use was obtained through data from industry related to sales figures of PPPs to amateur users. In the future, these data source from industry will be replaced by more complete data from the national government. In addition, the determination of the amount of PPPs used in seed treatment was difficult during this research as well.
